# Lactate from the tumor microenvironment - A key obstacle in NK cell-based immunotherapies

**DOI:** 10.3389/fimmu.2022.932055

**Published:** 2022-10-18

**Authors:** Marek Jedlička, Tereza Feglarová, Lucie Janstová, Marcela Hortová-Kohoutková, Jan Frič

**Affiliations:** ^1^ Department of Modern Immunotherapy, Institute of Hematology and Blood Transfusion, Prague, Czechia; ^2^ Faculty of Science, Charles University in Prague, Prague, Czechia; ^3^ Cellular and Molecular Immunoregulation Group, International Clinical Research Center of St. Anne´s University Hospital Brno, Brno, Czechia

**Keywords:** lactate, immunotherapy, NK cell, T cell, cytotoxicity, immunosuppression,, immunometabolism

## Abstract

Recent findings about the new roles of lactate have changed our understanding of this end product of glycolysis or fermentation that was once considered only a waste product. It is now well accepted that lactate acts as a signaling molecule and fuel source for cancer cells in a glucose-restricted environment. Moreover, lactate and lactate dehydrogenase are markers of poor prognosis of many cancers and regulate many functions of immune cells. The presence of lactate in the tumor microenvironment (TME) leads to polarization of the immunosuppressive phenotypes of dendritic cells and impairs the cytotoxic abilities of T cells and NK cells, and as such lactate is a major obstacle to immune-cell effector functions and the efficacy of cell-based immunotherapies. Emerging evidence suggests that lactate in the TME might be a novel therapeutic target to enhance the immunotherapeutic potential of cell-based therapies. This review describes our current understanding of the role of lactate in tumor biology, including its detrimental effects on cell-based immunotherapy in cancer. We also highlight how the role of lactate in the TME must be considered when producing cell therapies designed for adoptive transfer and describe how targeted modulation of lactate in the TME might boost immune-cell functions and positively impact cellular immunotherapy, with a focus on NK cell.

## Introduction

Lactate is the end product of anaerobic glycolysis when oxygen levels are insufficient. In addition, our understanding of lactate function has shifted over the last decade; now, lactate is also seen as an important signaling mediator both at the cellular level and the systemic level as a modulator of cell behavior in health and disease (1–3).

During cancer, rapidly proliferating tumor cells produce lactate at high concentrations as a waste molecule of anaerobic and aerobic glycolysis. The extracellular concentrations of lactate released by tumor cells can reach up to 40mM, which is approximately 20 times higher than levels in healthy blood or tissue (4, 5). This metabolic state, in which cells rely on glycolysis rather than on oxidative phosphorylation in an oxygen-rich environment, is known as the Warburg effect. The rapid gain of ATP in a case of sufficient glucose supply is not the only benefit of this metabolic program (6, 7), as other intermediates arising from glycolysis serve as building blocks in other metabolic pathways linked to cell proliferation and protein synthesis (8, 9). The Warburg effect, originally described in tumor cells (10), has recently been reported in many immune cell types such as T cells (11), macrophages (12) or natural killer (NK) cells (13).

Lactate production is tightly linked with the activity of the cytoplasmic enzyme lactate dehydrogenase (LDHA), which mediates the reduction of pyruvate to lactate as well as the oxidation of NADH to NAD^+^ (14). Lactate is then exported outside of the cytosol by one of the monocarboxylate transporters (MCT) (15, 16).

**Figure 1:**
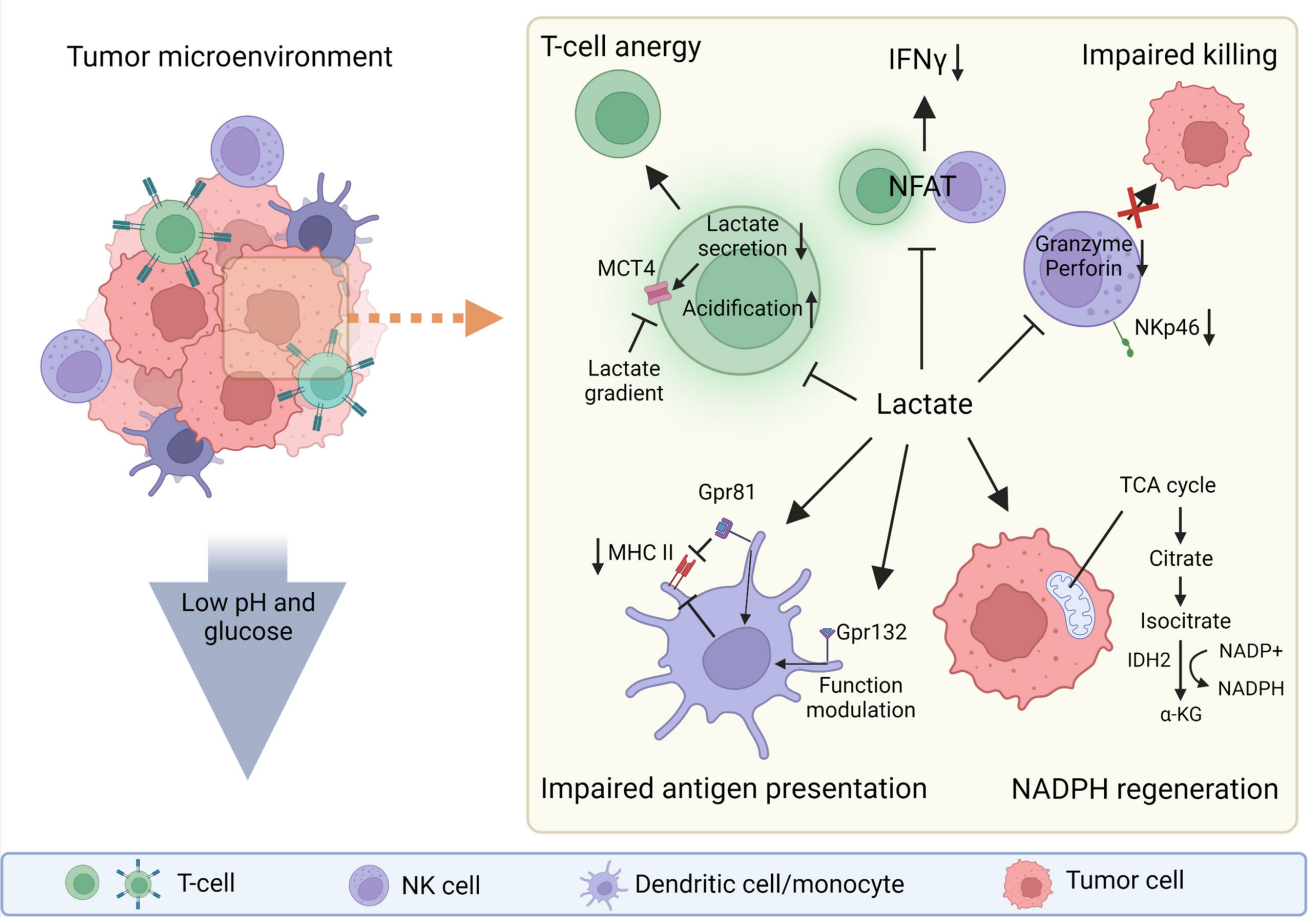
The roles of lactate in tumor microenvironment (TME). Lactate can be strongly immunosuppressive in the tumor microenvironment (TME) or can act as a molecule that helps tumor cells to regenerate NADPH and thus maintain redox balance and support their biosynthetic demands. The low pH and low glucose concentration in the TME also support the immunosuppressive function of the TME. Lactate directly or indirectly influences every cell in the TME. Gpr81, lactate receptor; MHC II, major histocompatibility complex class II. Created with BioRender.com.

Lactate not only serves as an end product of glycolysis, it is involved in NADPH production (**Figure 1**) in glucose-restricted environments, such as the one found in the tumor microenvironment (TME) (17). NADPH is crucial for maintaining redox balance (18) and reductive biosynthesis (19), which are key conditions for tumor growth. The cells activated through specific activation membrane receptors have increased energy uptake (20), which is linked with high lactate production. Lactic acid selectively disables activation of cytotoxic cells including NK cells and therefore impairs immune surveillance and possibly also the cytotoxic properties of therapeutic NK cells prepared for adoptive transfer (21). While many roles of lactate in solid tumors are broadly reported, the role of lactic acid in haemato-oncology disorders seems to be more nuanced. Nevertheless, increased levels of lactate and LDHA have been frequently reported as markers of poor prognosis in different types of leukemia (22, 23) such as acute lymphoblastic leukemia (ALL) (24, 25), acute myeloid leukemia (AML) (26–29), chronic lymphoblastic leukemia (CLL) (22) and chronic myeloid leukemia (CML) (30). Highly active tumor cells, as well as activated immune cells, utilize glucose and glutamine to produce ATP, and also catabolize lactate to produce NADPH.

Given the growing efforts to employ the cellular responses of NKs and CD8^+^ T cells in immunotherapies against tumor and leukemic cells, the connection between lactate metabolism and lactate levels and the control of the cytotoxic responses of T and NK cells is an area of intense study. Here we overview current knowledge of the role of lactic acid, particularly that derived from the TME, on the function of immune cells, with a focus on NK cells. We also highlight the remaining research gaps that need further study to gain a deeper understanding of lactate metabolism and improve the efficacy of immunotherapies driven by NK cells.

## The role of lactate in the tumor microenvironment

Robust evidence of the role of lactate in shaping the function of many immune cells has recently emerged (31–34). The ability of lactate to modulate the function of immune cells in the TME is well-described; in the TME lactate serves as a pro-tumorogenic molecule by inhibiting the function of effector cell types such as effector T cells (CD4^+^ (33), CD8^+^ (28, 35, 36)) and NK cells (34, 37) and supports the development of suppressor cells such as T regulatory cells (Treg) (32, 38), myeloid-derived suppressor cells (MDSCs) (37, 39) and tolerogenic dendritic cells (tDCs) (32).

Among several described mechanisms of lactate-mediated immunosuppression in the TME is the metabolically driven effect of lactate on T cells. Lactate limits the glycolytic flux of T cells and thus shifts them toward tolerance through several mechanisms. These mechanisms involve lactate accumulation resulting in decreased glyceraldehyde 3-phosphate dehydrogenase (GAPDH) activity. Because the activity of glycolytic enzymes favors the production of pro-inflammatory cytokines, the decrease in GAPDH activity limits their synthesis. Secondly, the lactate-rich environment does not limit Treg function and expansion, thus supporting the induction of tolerance (32, 38). In addition, lactate is required for intra-tumoral Tregs to support tumor progression (40). Lactate also suppresses the proliferation and function of cytotoxic (CD8^+^) T lymphocytes (CTLs) by selectively inhibiting p38 and c-Jun N-terminal kinase activity, resulting in reduced IFN-γ production (41). Lactic acid also impairs the recruitment of CTLs to the TME by blocking their motility. Hass et al. observed that chemotaxis of CD4^+^ and CD8^+^ T cells is reduced by differently expressed lactate transporters (Slc16a1 on CD8^+^ T cells and Slc5a12 on CD4^+^ T cells). The authors also showed that the lactate and glycolytic pathways are key regulators of chemokine-induced T-cell migration (35). Lactate diminishes the cytotoxicity of CTLs by lowering intracellular levels of perforin and granzyme B and reducing lytic granule exocytosis (35, 36, 42). Moreover, activated T cells in the TME not only have to compete with tumor cells for glucose, but must also cope with intracellular acidification resulting from tumor cells *via* MCT-mediated lactate transmission (43). High levels of extracellular tumor-derived lactate in the TME prevent activated T cells from secreting lactate into extracellular space due to the concentration gradient of lactate across the membrane. This gradient causes endogenous lactate to accumulate, which hampers the antitumor activity of effector T cells (1). The similar principles should apply in CD8^+^, CART and/or NK cells. Nevertheless, this field and comparison still lack consistent data, so our discussion remains rather speculative. Neutralization of the acidic TME and proton-pump inhibitors can reverse the suppression of antitumor immunity and improve immunotherapy (44).

Lactate also suppresses inflammasome assembly, lipopolysaccharide (LPS)-stimulated cytokine secretion and migration of macrophages and monocytes (39). The effects of lactate and the overall acidity of the TME are dependent on the lactate concentration. On one hand, lactate promote the differentiation of monocytes to dendritic cells (DCs) with an immunosuppressive phenotype by stabilizing HIF1α. On the other hand, high levels of lactate in the TME prevent the differentiation of monocytes to DCs (45, 46). Lactic acid suppresses the inflammatory functions of macrophages (M1-like) and enhances regulatory (M2-like) polarization, and these effects are dependent on MCT transport and HIF activation (47). Interestingly subsets of macrophages (M2-like) are able to directly monitor the levels of lactate through G protein-coupled receptor 132 and modulate its functions according the lactate presence (48). Similar to the inhibitory effects of lactic acid reported in monocytes and macrophages, lactic acid reduces DCs maturation and suppresses LPS-induced cytokine production (39). Lactic acid limits cell presentation of tumor antigens by activating G protein-coupled receptor 81 (Gpr81; a receptor for lactate) and inhibits the expression of major histocompatibility complex II (MHC-II) (49) (**Figure 1**). Moreover, lactic acid suppresses immunoglobulin (Ig)E- and IL-33-dependant inflammatory cytokine and chemokine production (50). In neutrophils, lactate induces the formation of neutrophil extracellular traps (51).

Since the production of lactate acidifies the TME, several studies have investigated the effects of an acid environment on cellular function. Lowering the pH from 6.8 to 6.0 in NK cells decreases in levels of granzyme B and perforin mRNA; furthermore, lactate may interfere with secretory pathways in NK cells and thus modulating the activity of the cytolytic machinery (15, 37). Exposure of NK cells *in vitro* to 15mM lactate, which is comparable to *in-vivo* concentrations in tumors (4, 52), lowered the expression of the NKp46 activation receptor (37) (**Figure 1**). However, the levels of lactate may vary based on type of the tumour (5, 52). Another study showed that lactic acid produced by colorectal cancer cells causes apoptosis of isolated liver resident NK cells *in vitro* and lowers the amount of tumor-infiltrating NK cells *in vivo (* 34). Brand and colleagues showed that the production of lactic acid by cancer cells limits CTL and NK cell activation (**Figure 1**) and impairs IFN-γ production; curiously this pattern was not present in CD4^+^ and IL-17^+^ CD4^+^ (Th17) T cells. Lactic acid-induced acidification also inhibits the transcription factor nuclear factor of activated T cells (NFAT), which results in decreased IFN-γ production (21) (**Figure 1**). The direct link between lactate levels and NFAT activity is an important finding, as NFAT plays a major role in orchestrating activities not only in T cells but also in other cell types including NK cells (53, 54). Interestingly lactate can influence cytosolic calcium (Ca^2+^) availability through pyruvate and α-ketoglutarate concentrations (55, 56). This could possibly influence NFAT function since it is regulated by Ca^2+^ abundance (54). The expression of LDHA negatively correlates with the survival of cancer patients, and reduces the numbers and activity of CD8^+^ T cells (21). Elevated lactate levels indirectly inhibit NK cell function by increasing the number of MDSCs (57). MDSCs are a heterogeneous population of immature myeloid cells that mediate the immunosuppressive environment in the TME. As well as suppressing NK cell activity, MDSCs prevent DCs maturation and inhibit T cell activation (37). The findings from the studies that have investigated influence of lactate on various immune cells need to be considered during further research leading to the use of adoptive transfer of NK cells to target solid tumors.

Another marker of poor prognosis in oncology patients is serum levels of LDH, as shown in CLL (22) and AML (24). One study suggested that tumors with elevated lactate dehydrogenase A (LDHA) levels are more prone to immune evasion, and therefore tumor progression occurs due to limited anti-tumor mechanisms (21). In contrast, another study showed that LDHA is crucial for the anti-tumor and anti-viral functions of murine NK cells (58). However, it is difficult to obtain data that directly indicates the role of lactate on the different mechanisms of NK cell-mediated killing in the TME. Since the environment is very complex and lactate influences every cell present, NK cell inhibition can occur due to many mechanisms. These mechanisms include the direct inhibitory effects of lactate on NK cells (34) or indirect effects, whereby lactate alters the function of other immune cells, which then inhibit the function of NK cells^,37,40^.

Thus, overcoming the negative effects of lactate on cell-based immunotherapies remains elusive. A study that used LDH isolated from various cell lines showed that millimolar concentrations of exogenous pyruvate can inhibit LDH function in an MCT-1 dependent manner (59). But since these experiments have not been performed in the TME, it can only be speculated that a similar mechanism occurs in the TME. Hence, this finding needs to be validated in the TME before pyruvate could eventually be used in a clinical setting. *In vivo* experiments that injected a melanoma cells into mice showed that LDHA knockdown decreases PD-L1 expression, and thus makes tumor cells more susceptible to anti-PD-1 treatment. Mice with LDHA knockdown also have higher infiltration of NK and CD8^+^ T cells into tumors. These findings stimulate further research, opening a space for the eventual pharmacological targeting of LDH (60). Furthermore, strategies that remove lactate from the TME are emerging (15, 16, 61). In recent years the various efforts to lactate removal appeared. They aim mainly to lactate export by blocking MCT (15, 16, 62), however also systemic alkalization (61) approaches or lactate traps (63) have been tested. Blocking MCT4 (the main transporter for lactate secretion) in cancer cells restores the cytotoxicity of NK cells that was inhibited by the lactate environment (16). Another approach showed that alkalization of the tissue milieu by oral administration of sodium bicarbonate in a mouse model of λ-myc lymphoma restores IFN-γ production by NK cells. However, this approach did not restore the cytotoxic functions of NK cells (61). Overall, lactate is an omnipresent substance in the TME, and as such it directly influences the outcome of anti-tumor immunity (37, 64, 65).

## Lactate is an obstacle to a successful immunotherapy

The production and export of lactate by rapidly proliferating tumor cells not only (8, 66) influences the function of immune cells and supports the ability of the tumor to grow and escape immune recognition (as discussed above); the acidification of the TME also limits the efficacy of cell-based immunotherapies (15, 45, 62, 67). The cells used for adoptive transfer thus need to be in the best possible fitness and metabolic state to be able to overcome TME-mediated inhibition (13, 16, 58). The findings of lactate interference with NFAT signaling possibly also impair not only IFNγ expression but also key NK cell maintenance cytokines as IL-2 as it is NFAT dependent (68, 69).

NK cells, the key cells responsible for cancer immunosurveillance, eliminate cancerous cells by a multistep process. First, the cancer cell is recognized through a series of specific activatory and inhibitory receptors that screen for the density of human leukocyte antigen (HLA) molecules and other damage markers on the surface of controlled cells. The eventual cytotoxic elimination of the target cells is then initiated by several mechanisms including integrin- or antibody-mediated adhesion of NK cells to the target cell, followed by the formation of an immunologic synapse and release of lytic granules (70–72). As we and others reviewed earlier, this process is energetically demanding and therefore tightly orchestrated to maintain the energetic homeostasis of organism (20, 73).

**Figure 2:**
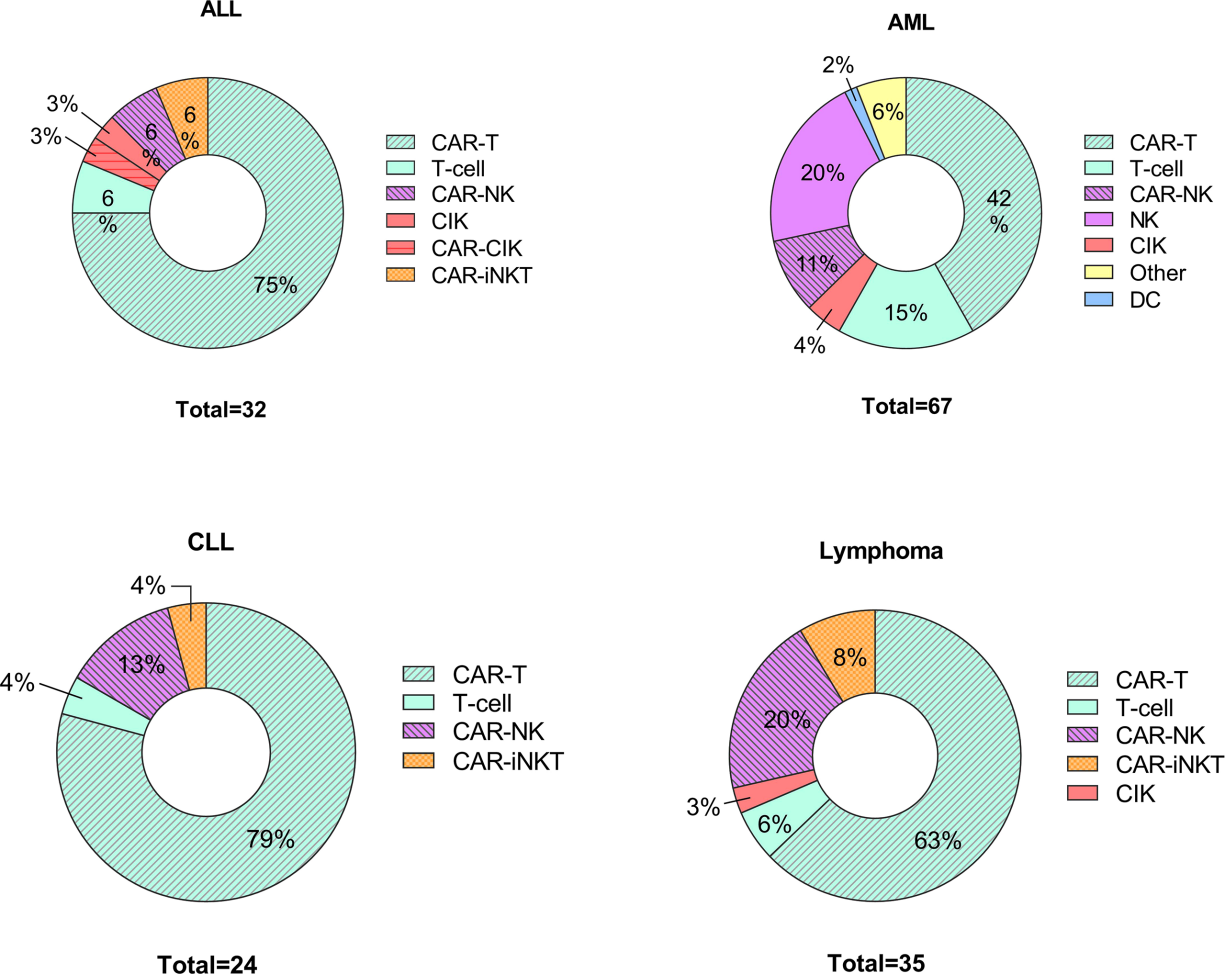
Cell-based therapies in hematological disorders. Graphical representations of clinical trials of adoptive cell transfer in hemato-oncological malignancies posted to *clintrials.gov* between 1^st^ of January 2020 – 1^st^ of March 2022. ALL, acute lymphoblastic leukemia; AML, acute myeloid leukemia including myelodysplastic syndrome; CLL, chronic lymphoblastic leukemia; CIK, cytokine induced killers; iNK, invariant NK cells.

Recent research has focused on developing new protocols for the use of adoptively transferred immune cells to treat various disorders including cancers (74–76) and autoimmune diseases (77, 78). Indeed immunotherapies that use different approaches and cell types are under investigation, including DC-based therapies, CAR T cells, NK cell-based therapies and CAR NK cells (75, 79–81). The efforts to treat various haemato-oncologic disorders using adoptively transferred immune cells are expanding; since 2020, there are 158 newly registered clinical studies (**Figure 2**).

One of the major benefits of the NK approach (compared with approaches that use other types of immune cells) is the partial prevention of graft versus host disease, as shown in preclinical studies (82, 83). The therapy setups include *ex vivo* cytokine-primed NKs (84–86), CAR NKs (79, 87, 88) and bi- or tri-specific killer engagers (BiKEs or TriKEs) (83, 89, 90). BiKEs and TriKEs are NK cells with two or three single-chain variable fragments, respectively, with various antigen specificity enabling precise cell-to-cell contact with tumor cell (91). A phase I/II clinical trial (NCT01904136) of an NK-based therapy showed promising results. The study aimed to decrease cancer relapse after a stem-cell transplant using *ex vivo* mb-IL21-expanded NK cells. The disease-free survival was 66% in patients who received multiple high doses of NK cells and 44% in controls, and the relapse rate was 9.5 times lower at 24-months follow-up (81). However, cells that are expanded in the optimal conditions of cell culture still have to face the strongly immunosuppressive niche of TME after adoptive transfer (92, 93). Since lactate is one of the most abundant metabolites in the TME (64, 94) its presence causes NFAT-regulated NK and CD8^+^ T cell suppression (21), impairment of NK cell cytolytic function (15, 34, 37) or inhibition of antigen presentation by DCs (49), which impairs the anti-tumor functions of these cell types (21, 45, 49, 95).

Intensive efforts have been invested into NK cell-based immunotherapy (96–98). The research field was boosted by the enormous success of several commercialized CAR T cell products. Improving the half-life of adoptively transferred cells and maintaining their cytotoxic capacity remain important research tasks. Efforts to improve these therapies drive research into a better molecular understanding of metabolic processes in T cells and NK cells, which closely control their cellular functions. Immunometabolism in the cytotoxic cells used for immunotherapy is essential to maintain their massive proliferation *ex vivo*, but the immunometabolism further changes after adoptive transfer as cytotoxic granules develop and the cells successfully adapt to their environment (13, 99, 100). NK cells are beneficial for both solid and hematological tumors (82, 101–103). Nersesian et al. examined 53 studies of various solid cancers and concluded that NK-cell infiltration into the tumor correlates with improved overall survival (102). Results from an experimental model of NK cell infiltration into tumor tissue support these clinical data. In these experiments, cancerous pancreatic cells were treated with an NK cell-recruiting protein-conjugated antibody (NRP-body), which after binding to the pancreatic tumor cell released a chemotactic molecule for NK cells. This boosted the ability of NK cells to infiltrate the tumor stroma and thus improved the outcome of immunotherapy (104). In hematological tumors, NK cell-based therapies benefit from a possible graft versus leukemia (GvL) effect by alloreactive NK cells (103, 105) and from the ability of NK cells to migrate into the bone marrow to eliminate leukemic cells (106). This latter feature of NK cells can be improved by incorporating chemokine receptors into the NK-based therapy that are specific for homing into the bone-marrow niche (107).

However, despite the initial success of NK cell-based therapies, the TME is one of the major obstacles to their success (92, 93). As well as lactate, transforming growth factor β (TGF-β) also suppresses immune cells in TME and other immune-suppressive molecules may be present (39, 108, 109). Nevertheless, studies show that NK cells expanded with IL-21-expressing feeder cells are not suppressed by TME in a model of ovarian cancer (13). Terren et al. showed that cytokines modulate the metabolism of NK cells and glycolysis is important for NK cell effector functions (86).

However, specific evidence about immunometabolism changes in adoptively transferred immunotherapeutic cells is scarce as it is difficult to obtain data. Therefore, the molecular mechanisms that orchestrate immunometabolism are highly underexplored. Adoptively transferred NK cells are dependent on the balance of activity of activator and inhibitory receptors and their cytotoxic machinery consisting of cytotoxic mediators including granzyme B and perforin. The success of adoptive transfer and further cytotoxicity are tightly dependent on NK cell status, fitness and donor variability (110). Therefore, the presence of lactate in the TME must be taken into account during the production of immunotherapeutic cells.

## Conclusions and future perspectives

The functions of lactate, originally described and long-understood as an end product of metabolism, have recently been intensively studied and completely reconsidered. The plethora of roles of lactate now includes cancer biomarkers and target molecules for therapies. Together with LDHA, lactate is a marker of poor prognosis in haemato-oncological patients (23, 24, 62). As one of the main inhibitory molecules produced in the TME, lactate is a crucial obstacle to a patient´s immune response or the efficacy of cell-based immunotherapies in various cancers (92, 93). Lactate can trigger signaling in immune cells and thus limit their effector functions. These recent findings open the door to lactate targeting to boost the immune response to cancer. To date, lactate has been targeted almost exclusively by blocking its secretion from tumor cells, and this strategy shows promising results in some cancers (16, 62). However, with the advent of cell-based immunotherapies (NK cells, T cells), new questions need to be addressed. Contemporary research accepts the crucial role of metabolism in the function of immune effector cells (73), and so efforts are underway to try to modulate immunometabolism to produce superior therapeutic cells (73, 99). Nevertheless, this approach is still in its infancy and needs further research (20, 73, 111). In order to improve the outcome of NK cell-based therapies, the deeper understanding of NK cell cytotoxicity in patient´s body is needed. We also need to better understand the trajectories of therapeutic NK cells after adoptive transfer. This knowledge could be reached with the use of new models of bone marrow or tumor niche microenvironmnet. Furthermore, the precise map of NK cell metabolism might lead to possibility of precise metabolic-based modification during expansion and preparation of therapeutic cells in order to overcome inhibitory niche of TME. To conclude, even though new protocols to enhance the immunotherapeutic potential of cell-based therapies are emerging, lactate still plays an important role in thwarting of success of these approaches and so is a key obstacle to better cancer treatment.

## Author contributions

MJ wrote and drafted the manuscript. TF, MH-K, and JF reviewed and edited the manuscript. LJ create the figures. All authors contributed to the article and approved the submitted version.

## Funding

The authors were supported by the Ministry of Health of the Czech Republic grant nr. – NU22-08-00287 all rights reserved and DRO (Institute of Hematology and Blood Transfusion – IHBT, 00023736) and by the European Social Fund and European Regional Development Fund – Project MAGNET (No. CZ.02.1.01/0.0/0.0/15_003/0000492) and ENOCH (CZ.02.1.01/0.0/0.0/16_019/0000868).

## Acknowledgments

Authors would like to thank Dr. Jessica Tamanini from Insight Editing London for critically reviewing the manuscript before submission.

## Conflict of interest

The authors declare that the research was conducted in the absence of any commercial or financial relationships that could be construed as a potential conflict of interest.

## Publisher’s note

All claims expressed in this article are solely those of the authors and do not necessarily represent those of their affiliated organizations, or those of the publisher, the editors and the reviewers. Any product that may be evaluated in this article, or claim that may be made by its manufacturer, is not guaranteed or endorsed by the publisher.
